# A remineralizing orthodontic etchant that utilizes calcium phosphate ion clusters

**DOI:** 10.3389/fbioe.2022.944869

**Published:** 2022-08-31

**Authors:** Hyeryeong Kim, Kyung-Hyeon Yoo, Seog-Young Yoon, Youn-Kyung Choi, Yong-Il Kim

**Affiliations:** ^1^ Department of Orthodontics, Dental Research Institute, Pusan National University Dental Hospital, Yangsan, South Korea; ^2^ School of Materials Science and Engineering, Pusan National University, Busan, South Korea; ^3^ Department of Orthodontics, Biomedical Research Institute, Pusan National University Hospital, Busan, South Korea; ^4^ Dental and Life Science Institute, School of Dentistry, Pusan National University, Yangsan, South Korea

**Keywords:** calcium phosphate ion clusters (CPICs), phosphoric acid, etchant, self-adhesive resin, bracket debonding, enamel bonding, enamel remineralization

## Abstract

This study aimed to investigate whether a phosphoric acid (H_3_PO_4_) solution containing calcium phosphate ion clusters (CPICs) could minimize enamel damage during long-term bracket bonding by dissolving the enamel surface and promoting enamel remineralization. The experimental design is as follows: first, three experimental etchants (H_3_PO_4_, CPICs-incorporated H_3_PO_4_ solution-I, and CPICs-incorporated H_3_PO_4_ solution-II) and two bonding resins (conventional orthodontic resin and self-adhesive orthodontic resin) were used in combination to create six groups, respectively. Each of these six groups was then divided into two sub-groups based on the presence or absence of thermocycling (TC). Twenty samples were assigned to each of the 12 groups (independent variables), and thus a total of 240 metal bracket-attached human premolars were used in this experiment. Bracket debonding was performed on each of 20 premolars in 12 groups, and shear bond strength (SBS) and adhesive remnant index (ARI) values were measured as dependent variables. Next, the three experimental etchants were applied (independent variables) to each of the three enamel samples, and the remineralization of the enamel surface was investigated as a dependent variable. The enamel surface was observed using electron scanning and atomic force microscopy. Furthermore, X-ray diffraction, energy dispersive spectroscopy (EDX) spectrum X-ray spectroscopy, and elemental mapping were performed, and the Knoop microhardness scale was measured. Therefore, the experiment was performed in two steps: SBS and ARI measurements for 12 groups, followed by observation of the enamel surface and microhardness measurements, according to the three types of etchants. As a result of the experiment, first, when the bracket was debonded, SBS did not decrease, and residual adhesive was hardly observed in the C2A group (before TC), C2A, and C1C groups (after TC) (*p* < 0.001). Second, the experimental etchant containing CPICs achieved remineralization while demineralizing the enamel. This was verified through SEM/EDX, element mapping, XRD, and AFM. Also, the roughness and microhardness of the enamel surface were better in the remineralized surface by the experimental etchant containing CPICs (*p* < 0.017). The CPICs-incorporated H_3_PO_4_ solution reduced ARI while maintaining SBS during bracket debonding, regardless of whether TC was performed or the type of resin. The etchant containing CPICs was also shown to remineralize the enamel and increase its microhardness.

## 1 Introduction

In orthodontic treatments, the bonding of orthodontic brackets is an essential process; however, it is also a temporal procedure, as the brackets must be removed at the end of the treatment. This requires that the shear bond strength (SBS) be sufficiently strong to prevent detachment, but sufficiently weak to prevent damage to the enamel layer when detached. After the bracket is removed, the enamel layer should be undamaged and the adhesive residue should be minimal. Recent research has focused on reducing the number of bracket attachment process while increasing its durability (Latta and Radniecki, 2020). For example, self-adhesive resin (SAR) is a product that does not use an adhesive, and because it reduces the bracket attachment process by one-step, it can help to reduce the overall level of adhesive residue (Cengiz and Ünal, 2019; Iglesias et al., 2020; Rasmussen et al., 2020; Ok et al., 2021).

However, since the development of the total etching technique, there have been continuous studies showing that the etch-and-rinse system is rather aggressive on the enamel when used for orthodontic bracket attachment. There are several iatrogenic factors for acid etchants, such as a rough surface and increased surface porosity due to excessive etching, susceptibility to cavities due to the loss of a fluoride-rich layer, fracture, and cracks during bracket debonding (Yadav et al., 2013). Therefore, there was an effort to develop a “mild” etchant that could replace the 35% phosphoric acid used previously.

Ajaj et al. (2020) reported that the remineralization of white spot lesions resulting from orthodontic treatments could be better improved when using a mild etchant containing 10% polyacrylic acid than using H_3_PO_4_. This is because smaller pores were formed by the formation of a larger number of pores when compared with H_3_PO_4_. Furthermore, Fu et al. (2004)used X-ray diffraction (XRD) data to show that the reaction between 15% maleic acid and HA forms calcium maleate and that the byproducts were calcium hydrogen phosphate [Ca(H_2_PO_4_)2H_2_O] and calcium hydrogen phosphate hydrate [CaHPO_4_(H_2_O)_2_]. In other words, maleic acid can be used as a functional component of a self-etching primer by chemically bonding to the enamel surface *via* an ionic bond.

Efforts have been made to overcome these iatrogenic factors while preventing the loss of minerals: remineralizing or preventing demineralization by supplying fluoride (Jablonski-Momeni et al., 2020; Aldhaian et al., 2021; Althagafi, 2022), including components for remineralization in pretreatment materials such as adhesives (Liu et al., 2018; Wang et al., 2018; Yang et al., 2022), processing adhesives prior to pretreatment of remineralizing materials such as casein phosphopeptide-amorphous Ca–P (ACP) or bioactive glass (Ladhe et al., 2014; Mobarak et al., 2015; Bakry and Abbassy, 2019; Abbassy et al., 2021; Aref and Alrasheed, 2022), and the use of Ca or Ca–P-based materials in the enamel etching solution (Ibrahim et al., 2019; Ibrahim et al., 2020; Tuma and Yassir, 2021). Ibrahim et al. (2019) reported the development of an etchant paste prepared by mixing β-tricalcium phosphate and monocalcium phosphate monohydrate powder with 35% H_3_PO_4_. This solution was found to facilitate enamel adhesion and has the potential to remineralize during the post-debonding step. Cruz et al. also published a “new enamel-protective material” that promotes bracket adhesion and induces the formation of Ca–P crystals on the enamel surface (Cruz-González and Delgado-Mejía, 2020). However, the study of Cruze et al. could not provide quantitative evidence other than SEM data for the formation of Ca–P crystals. In the study of Ibrahim et al. (2019), X-ray diffraction (XRD) and micro-Raman spectroscopy results were presented as quantitative evidence for remineralization, but more evidence are needed other than these two studies. Unfortunately, there have been few studies so far confirming whether remineralization by including a Ca–P-based material in the etchant. It is not easy to clearly demonstrate the role of Ca-based materials in etchants based on the limited literature.

A novel material called calcium phosphate ion clusters (CPICs) was published under the title of the “biomimetic mineralization frontier” in 2019 (Shao et al., 2019). Later, Kim et al. reported that when CPICs were used in dentin-resin bonding pretreatments, the CPICs inhibited matrix metalloproteinases (MMPs) using an ethanol wet-bonding mechanism, and this increased adhesion to the resin by remineralizing dentin. As a follow-up study, Kim et al. (2020) tried to investigate whether remineralization occurs while acting as an etchant when CPICs are incorporated in the 35% H_3_PO_4_ etchant and treated on enamel. This idea is the beginning of this research, and no research has been performed on enamel treated with CPICs-incorporated 35% H_3_PO_4_.

On the other hand, the research team conducted a study in which CPICs-incorporated 35% H_3_PO_4_ was also treated in dentin. As a result, Lee et al. (2022) recently demonstrated that the CPICs-incorporated 35% H_3_PO_4_ acts as an etchant and increases adhesion with the resin by remineralizing the dentin-resin hybrid layer. Based on these studies, a 35% H_3_PO_4_ solution containing CPICs was applied in this investigation to enamel to determine whether it acts as an etchant and at the same time to affect enamel remineralization during the long period of orthodontic bracket attachment. Until now, there have been few studies on whether remineralization occurs when an etchant contains a Ca–P-based material and is treated on teeth. This is the first study to treat enamel by including CPICs in the etchant and will become a cornerstone for the development of new orthodontic or conservative etchants in the future.

This study aimed to investigate whether enamel damage can be minimized during bracket debonding. If nanometer-sized CPICs can affect the bonding strength of orthodontic brackets and prevent enamel damage through enamel remineralization, this study could serve as a basis for the development of new orthodontic etchants and remineralized therapeutic etchants. The null hypothesis of this study was as follows: (Latta and Radniecki, 2020) a 35% H_3_PO_4_ solution containing CPICs would decrease the bonding strength of the bracket before and after thermal cycling and increase the adhesive residue; (Cengiz and Ünal, 2019) a 35% H_3_PO_4_ solution with CPICs would not remineralize the enamel; and (Rasmussen et al., 2020) 35% H_3_PO_4_ solution with CPICs did not increase the microhardness of the enamel surface.

## 2 Materials and methods

### 2.1 Overview

**FIGURE 1:**
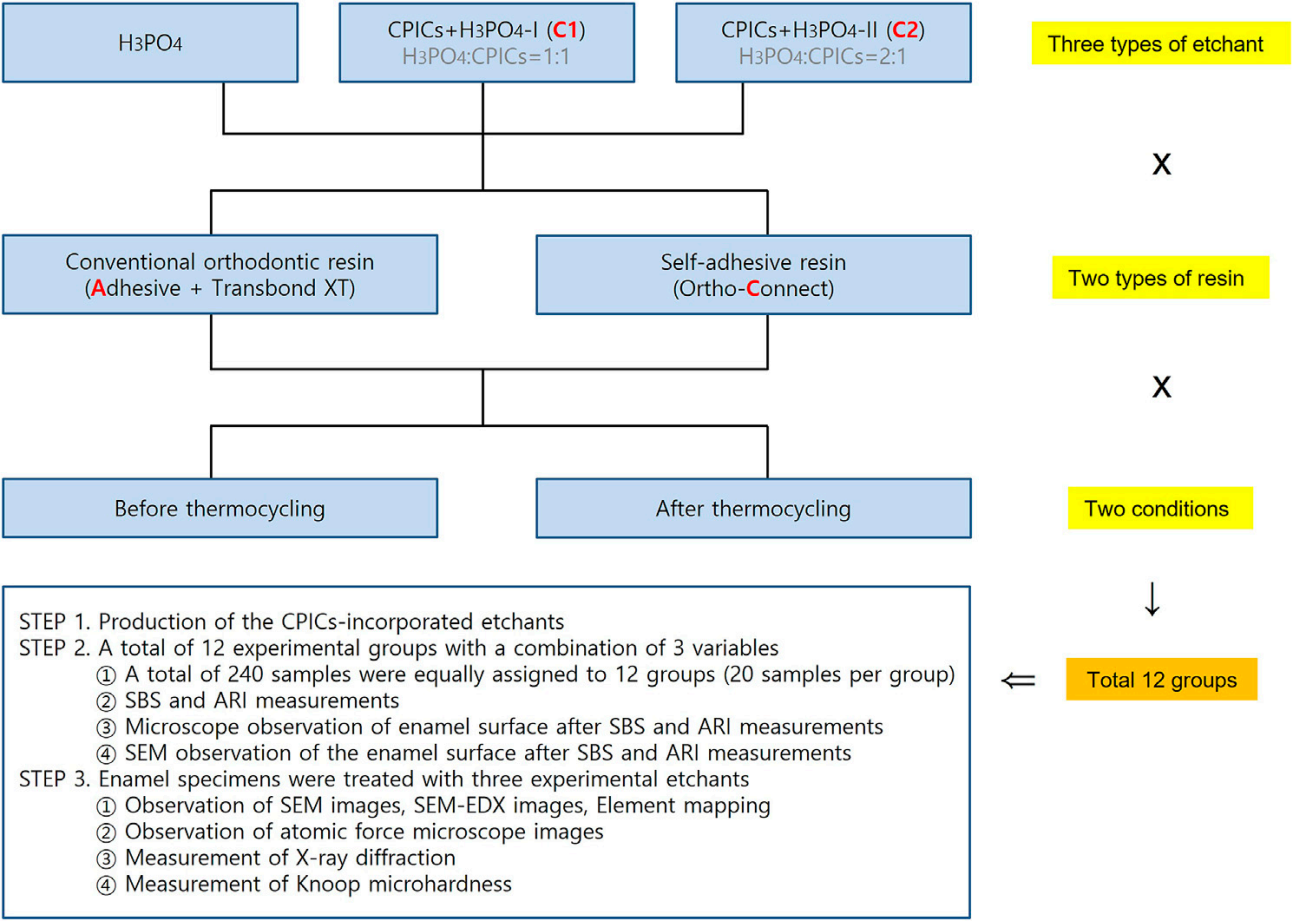
Experimental group design. Groups A and C were control groups that used pure 35% H_3_PO_4_ without CPICs. Group A used adhesive and Transbond XT resin, whereas Group C used Ortho Connect self-adhesive resin. C1 and C2 were experimental groups that used CPIC-incorporated in H_3_PO_4_ at a 1:1 (CPICs + H_3_PO_4_-I) and 1:2 (CPICs + H_3_PO_4_-II) volume ratios, respectively. Thus, the CPIC content of C2 was lower than that of C1. Three types of etchants (according to the inclusion and proportion of CPICs), two types of resins, and two conditions for thermocycling were used; thus, the experiment included 12 groups in total (H_3_PO_4_, phosphoric acid; CPICs, calcium phosphate ion clusters).

Three parameters were applied in this experiment: three types of etchants (H_3_PO_4_, CPICs + H_3_PO_4_-I, and CPICs + H_3_PO_4_-II), two types of resin (conventional orthodontic resin and self-adhesive resin), and the presence or absence of thermocycling, with 12 groups been created using variable combinations. Twelve experimental groups were set up using three designs, and 20 samples were assigned to each group, and thus a total of 240 metal bracket-attached human premolars were used (Figure 1). The experiment was conducted in three stages. STEP 1 was to make CPICs-incorporated etchant. In STEP 2, three experiments were conducted with the 12 groups. First, the SBS and adhesive remnant index (ARI) were measured during bracket debonding before and after thermocycling. Second, the enamel surface where the bracket fell off was observed using a medical optical microscope, and the same area was observed using a scanning electron microscope (SEM). In STEP 2, the independent variable is a combination of three variables and 12 groups with brackets attached, and the dependent variable is SBS, ARI, and microscopic images of the enamel surface from which the brackets have been removed. In STEP 3, three types of etchants (i.e., three groups) were applied to the enamel to determine whether remineralization occurred, and the resulting hardness of the enamel surface was assessed. Six experimental techniques were used in this study. First, the surface shape and roughness were observed by the SEM and atomic force microscopy (AFM), and XRD and EDX analyzes were performed to prove remineralization. After etching, the elemental mapping of Ca, P, and O on the enamel surface was performed, followed by a Knoop microhardness test. In STEP 3, the independent variable is the type of three etching solutions, and the dependent variable is the remineralization and hardness of the enamel surface, which is proven by six experimental techniques.

Again, the design of the experiments is simple: first, perform SBS 20 times in each of the 12 groups. Then, measure the ARI by observing the enamel part where the bracket has fallen. That is, SBS and ARI were measured with the same sample. Second, three etchant groups were made four times, and seven experimental data were obtained. The sequence is as follows: 1) SEM, SEM/EDX, and element mapping could be completed at the same time because the imaging was possible at the same time. 2) After AFM imaging, it was able to prepare a table by obtaining roughness data. Both data were obtained from the same sample. 3) XRD measurement. 4) Knoop hardness measurement.

There are many abbreviations used in this text. C1, that is, CPICs + H_3_PO_4_-I indicates that H_3_PO_4_ and the CPICs are mixed at a ratio of 1:1, and C2, that is, CPICs + H_3_PO_4_-II indicates that H_3_PO_4_ and the CPICs are mixed at a ratio of 2:1. The Roman letters I and II may be confused, leading to a misunderstanding that CPICs + H_3_PO_4_-II has a high proportion of CPICs, but the ratio of CPICs was higher in CPICs + H_3_PO_4_-I than in CPICs + H_3_PO_4_-II; the experimental results for STEP 3 are arranged in the order of the CPICs concentration gradient (H_3_PO_4_, CPICs + H_3_PO_4_-II, and CPICs + H_3_PO_4_-I).

### 2.2 Consumables

Unless otherwise specified, the following chemicals were used without further purification: calcium chloride dihydrate (CaCl2.2H2O; 99.0%) (Sigma-Aldrich, St. Louis, MO, United States), triethylamine [TEA (C2H5)3N; 99.5%] (Sigma-Aldrich, St. Louis, MO, United States), H_3_PO_4_ (85% solution in water, Sigma-Aldrich, St. Louis, MO, United States), ethanol (99.9%), etchant (35% H_3_PO_4_ solution; Ultra Etch, Ultradent, South Jordan, UT, United States), adhesive (Adper Scotchbond Multi-Purpose Plus, 3M, Monrovia, CA, United States), GC Ortho Connect (GC America, Alsip, Illinois, United States), Transbond XT (3M Unitek, Monrovia, California, United States), and metal brackets (YES2; Osstem Orthodontics, Seoul, Korea). Deionized water was used in the experiment, and all solutions were filtered through 0.22-µm Millipore films (MF-Millipore Membrane Filter, 0.22-µm pore size, Merck KGaA, Darmstadt, Germany) before use.

### 2.3 Trial design and blindness

The choice of experimental teeth did not perform any special procedure or randomization. Although randomization in a strict sense was not performed, it was determined that the bias could be minimized because the researchers tested the teeth without knowing individual information by pooling the teeth. For extracted teeth, the patient’s age range and healthy teeth (extraction for orthodontic teeth) were selected, so the baseline difference was not significant, and the researcher could not be completely blind. This is because the dental materials and procedures used in the experiment were different in each group. However, since each group was coded in the mechanical test and data analysis of the specimen, the bias was minimized. Each code used in the experiment is as follows: C1, CPICs + H_3_PO_4_-I; C2, CPICs + H_3_PO_4_-II; A, 35% H_3_PO_4_ + conventional orthodontic resin; C, 35% H_3_PO_4_ + self-adhesive orthodontic resin; C1A, CPICs-incorporated H_3_PO_4_ solution-I + conventional orthodontic resin; C2A, CPICs-incorporated H_3_PO_4_ solution-II + conventional orthodontic resin; C1C, CPICs-incorporated H_3_PO_4_ solution-I + self-adhesive orthodontic resin; and C2C, CPICs-incorporated H_3_PO_4_ solution-II + self-adhesive orthodontic resin.

### 2.4 Phase I: preparation of calcium phosphate ion clusters-incorporated H_3_PO_4_ solutions

**FIGURE 2:**
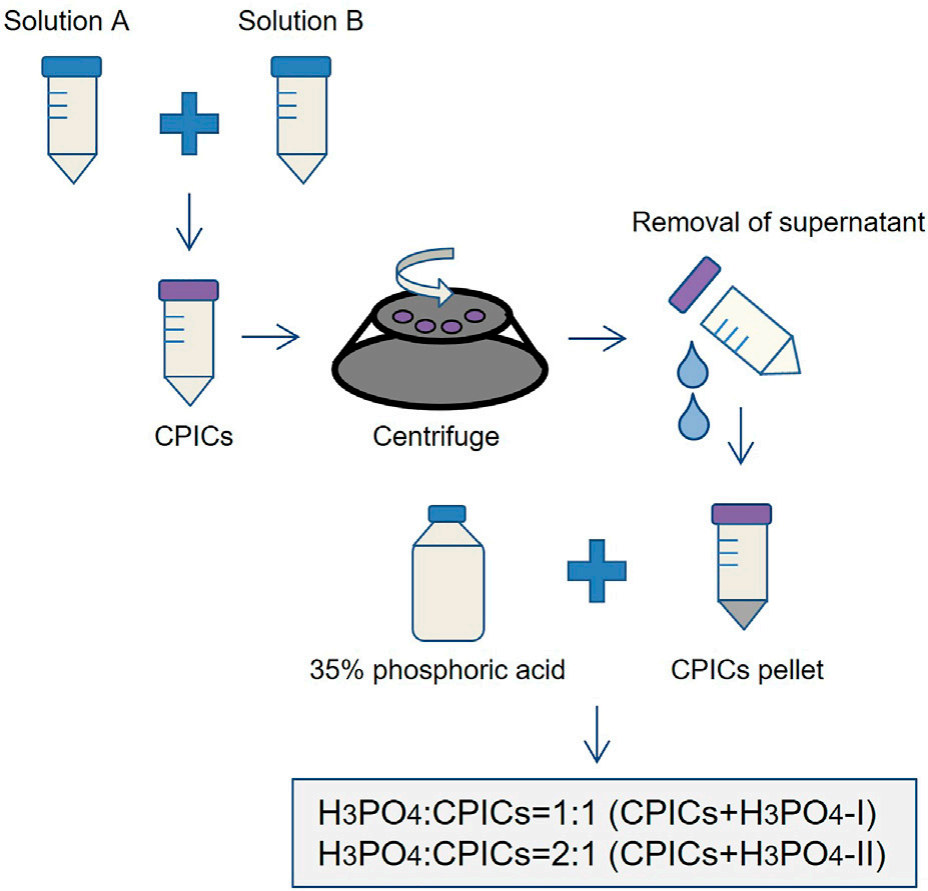
Experimental design of the etchants used in this study (CPICs, calcium phosphate ion clusters; H_3_PO_4_, phosphoric acid).

The CPICs-incorporated in the H_3_PO_4_ solutions were synthesized according to previously described methods (Shao et al., 2019). Briefly, solution A contained 80 ml of ethanol, 0.20 g of calcium chloride dehydrate, and 3.8 ml of TEA and was ultrasonicated (BRANSON, Danbury, CT, United States) for 5 min. Solution B contained 20 ml of ethanol and 70 µL of H_3_PO_4_ and was thoroughly stirred. Subsequently, solution B was added to solution A with slight agitation, and 2 mg/ml of CPICs were formed in solution. The ethanol-based CPICs solution was centrifuged at 3,000 rpm for 10 min at 4°C. After removing the supernatant portion, the remaining CPICs were mixed with 35% H_3_PO_4_ at 1:1 and 1:2 volume ratios, referred to as CPICs + H_3_PO_4_-I and CPICs + H_3_PO_4_-II, respectively. The negative control used was a 35% H_3_PO_4_ solution (indicated as H_3_PO_4_) (Figure 2). In this step, an etchant for experiments from Phase II to Phase VI is prepared. There were three types of etching solution used in the experiment: 35% H_3_PO_4_ (negative control), CPICs + H_3_PO_4_-II, and CPICs + H_3_PO_4_-I. Except for H_3_PO_4_, which is a negative control, the reason for setting up two experimental etchants is that there was no study using CPICs mixed with H_3_PO_4_ in a volume ratio. When the ratio of CPICs became more than 1/2 of the total, we were concerned that the etching ability would decrease, so the experiment was set up with 1/2 and 1/3 ratios of CPICs.

### 2.5 Phase II: enamel treatment with the experimental etchants, resins, and bracket bonding

Sound human premolars (no caries, previous restorations, or cracks) were used in this study. Extracted human premolars were collected from patients to carry out *in vitro* procedures after obtaining ethical approval from the ethical committee of Pusan National University Dental Hospital (PNUDH-2021-012).

In total, 240 metal bracket (YES2, Osstem Orthodontics, Seoul, Korea)-bonded human premolars were randomly allocated into six groups (*n* = 40 each), according to the bonding resin type combinations (conventional orthodontic bonding resin or the SAR) and etchants (35% H_3_PO_4_, CPICs + H_3_PO_4_-I, or CPICs + H_3_PO_4_-II) used. The six groups in this study were as follows: A, 35% H_3_PO_4_ + conventional orthodontic resin; C, 35% H_3_PO_4_ + SAR; C1A, CPICs + H_3_PO_4_-I + conventional orthodontic resin; C2A, CPICs + H_3_PO_4_-II + conventional orthodontic resin; C1C, CPICs + H_3_PO_4_-I + SAR; and C2C, CPICs + H_3_PO_4_-II + SAR (Figure 1).

To bond the brackets to the teeth, three kinds of etchants (H_3_PO_4_, CPICs + H_3_PO_4_-I, or CPICs + H_3_PO_4_-II) were applied for acid etching of the teeth for 30 s, followed by rinsing and drying. After visually confirming the chalky surface of the tooth after etching, the adhesive was applied. In all six groups, the experimental orthodontic bonding resins were applied to the bracket base corresponding to the long axis of the tooth. The excess resin was removed, and each resin was light-cured for 20 s. After bracket bonding, samples were stored in distilled water at 37°C for 24 h. Subsequently, half of the samples (*n* = 20 per group) were debonded to the bracket. The other half were subjected to a thermocycling (TC) regimen of 10,000 cycles between cold and hot water baths at 5 and 55°C, respectively, with a dwell time of 30 s in each bath and a transfer time of 5 s (ISO/TS 11405:2015, testing of adhesion to tooth structure), followed by bracket debonding.

At this stage, the number of groups is twelve: metal brackets were attached to the premolars using three types of etchants and two types of resins prepared in Section 2.3, and they were further divided into two groups, according to whether TC was performed. Therefore, it was 3 × 2 × 2 = 12 in total (Figure 1). There were 20 premolars with brackets assigned to each group. For estimating the sample size, power calculation was not separately used, and 20 samples per group were set up with reference to Ibrahim et al. (2019).

### 2.6 Phase III: testing shear bond strength and scoring adhesive remnant index before and after thermocycling

The SBS was measured using a universal testing machine (Instron, Canton, Massachusetts, United States) and calculated by measuring the maximum load (unit: Newton) with the crosshead at a speed of 1 mm/min divided by the bracket base surface area (3.3 × 3.6 mm). The resin remaining on the teeth was evaluated using the adhesive remnant index (ARI) score (Ibrahim et al., 2019) and then observed under a medical microscope (SG/M320 F12, Leica Microsystems, Singapore) and a scanning electron microscope (SEM; JSM-7900F; JEOL, Tokyo, Japan).

Phase III is a step to measure SBS at the moment the bracket is detached for the 12 groups prepared in Phase II. In 240 premolar samples for which SBS was measured, the amount of residual adhesive (ARI) on the enamel surface from which the bracket was removed scored in 0–3 steps. Next, the surface was observed with a medical microscope, and SEM imaging was performed.

### 2.7 Phase IV: analysis by scanning electron microscope imaging, scanning electron microscope/EDX, elemental mapping, and X-ray diffraction

The enamel surface was treated with experimental etchants for scanning electron microscopy, microhardness, and atomic force microscopy observations. To examine the enamel surface after the use of the experimental etchants, the crown of each tooth was sectioned mesiodistally through the occlusal central fossae using a low-speed diamond saw (Accutom-100; Struers Inc. Cleveland, Ohio, United States), with continuous irrigation. The buccal halves were embedded in an acrylic resin. The enamel surface was polished using a rotating polishing machine (1,200, 2,400, and 4,000 grit MetaServ 250; Buehler, Lake Bluff, Illinois, United States). The samples were then cleaned using water-bath ultrasonication. The experimental etchants (35% H_3_PO_4_, CPICs + H_3_PO_4_-II and CPICs + H_3_PO_4_-I) were applied to the buccal surfaces for 30 s, followed by irrigation with water and drying for 20 s each.

All samples were maintained dry under ambient laboratory conditions for 24 h, followed by scanning electron microscopy (SEM; JSM-7900F; JEOL, Tokyo, Japan). Moreover, the morphology and elemental chemical composition of the surface of the investigated samples were examined by scanning electron microscopy coupled with energy dispersive X-ray spectroscopy (SEM/EDX) under low vacuum conditions. In addition, to identifying the phases of the mineralized byproduct, X-ray diffraction analysis was performed using a diffractometer (XRD, Rigaku Ultima IV, Tokyo, Japan) with Cu Kα radiation (*λ* = 1.5406 Å). XRD patterns were recorded at a scan rate of 2 /min from 3 to 90.

### 2.8 Phase V: testing Knoop microhardness

Microhardness (MVK‐H1, Mitutoyo, Kanagawa, Japan) was determined under a 200 g force load and dwell time of 5 s, applying its indenter on top of the enamel surface. The measurements were repeated thrice.

### 2.9 Phase VI: measuring enamel roughness by atomic force microscopy

The enamel surface roughness of the samples was evaluated using atomic force microscopy (AFM) (Dimension Icon, BRUKER, Carteret, New Jersey, United States). This instrument was supported by a scanner with a maximum range of 50 μm × 50 μm × 10 µm in the x, y, and z directions, respectively. To measure the roughness values, the tip was moved across the surface, and 20 different points were measured for each group. These measurements involved three roughness parameters, expressed in nanometers. The average roughness (Ra) is the arithmetic mean of the height of the peaks and depth of the valleys from a mean line. The root-mean-square roughness (Rq) is the height distribution relative to the mean line. The maximum roughness depth (Rmax) represents the isolated profile features.

In Phase IV, V, and VI, three types of etchants were applied to the enamel surface embedded in acrylic resin, and the remineralization and hardness of the surface were measured. The number of groups at this stage is three: 35% H_3_PO_4_, CPICs + H_3_PO_4_-II, and CPICs + H_3_PO_4_-I. Three etchant groups were made four times, and seven experimental data were obtained. The sequence is as follows: 1) SEM, SEM/EDX, and element mapping could be completed at the same time because the imaging was possible at the same time. 2) After AFM imaging, it was able to prepare a table by obtaining roughness data. Both data were obtained from the same sample. 3) XRD measurement. 4) Knoop hardness measurement.

### 2.10 Statistical analysis

We evaluated the normality of the distributions of all continuous variables using the Shapiro-Wilk method. All SBS values after TC followed a normal distribution, but the remaining variables, including SBS values before TC, values for enamel roughness, and microhardness, were not normally distributed. Therefore, for SBS values after TC following a normal distribution, one-way analysis of variance (ANOVA) test and the Tukey’s honestly significant difference *post hoc* analysis were performed. For non-parametric variables, SBS values before TC, ARI values before and after TC, and values for enamel roughness and microhardness, Kruskal–Wallis test were performed. *p*-values for *post hoc* analysis of Kruskal–Wallis test was adjusted to remove type I error. Adjusted *p*-value was calculated by Mann–Whitney test with Bonferroni correction. The SBS and ARI values consisting of six groups multiplied the *p*-value by 15, and the roughness and microhardness values consisting of three groups were derived by multiplying the *p*-value by 3. We recorded the adjusted *p*-value in Tables 2, 4. The significance level at adjusted *p*-value was 0.05. All data were analyzed by using SPSS Statistics for Windows, Version 22.0 (IBM SPSS, Version 22.0, Armonk, NY: IBM Corp).

## 3 Results

### 3.1 Phase I: enamel etching and remineralization with CPICs + H3PO4-I and CPICs + H3PO4-II solutions

**FIGURE 3:**
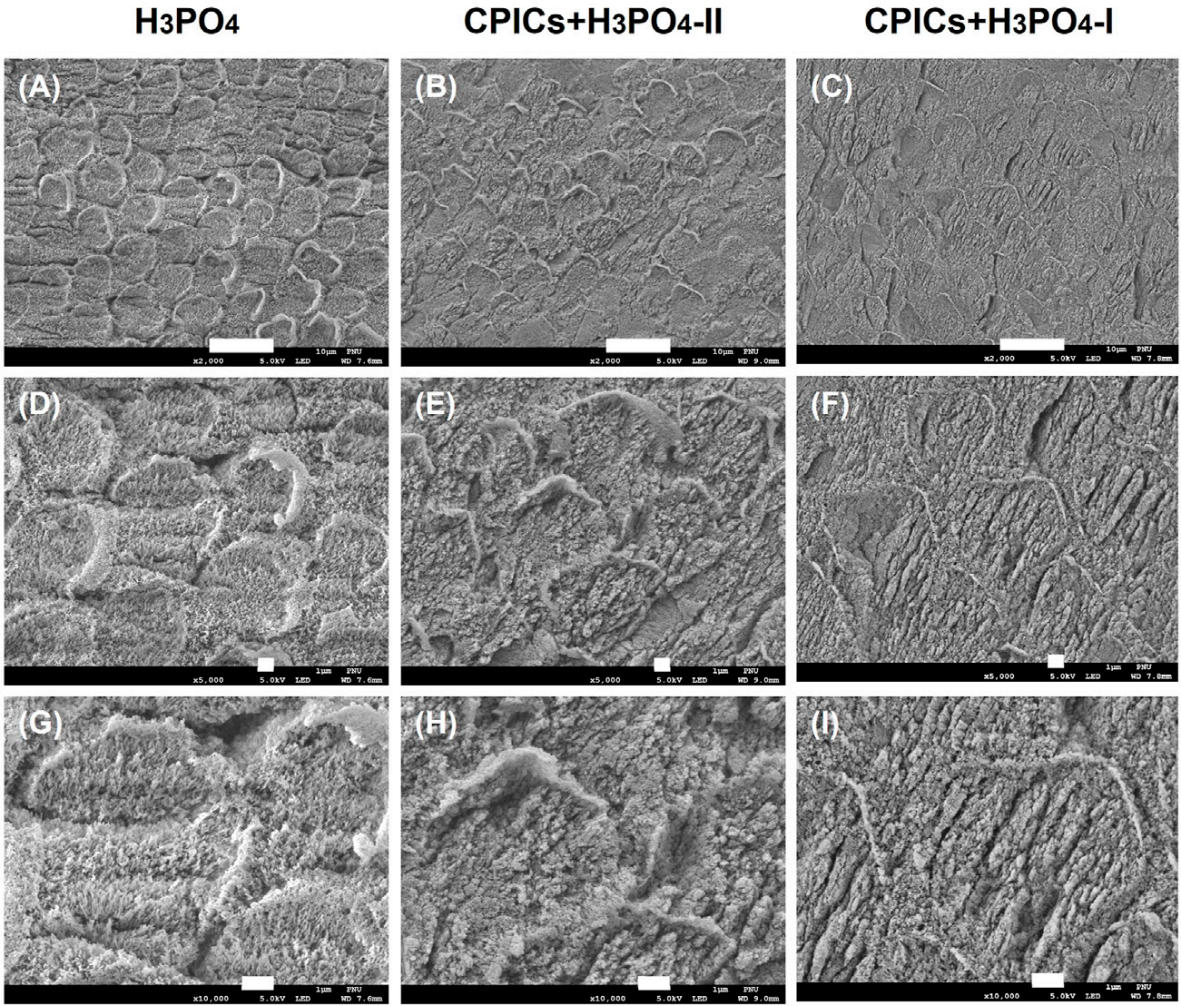
Scanning electron microscope images of the acid-etched enamel. Each row represents a different magnification (2,000×; 5,000×; and 10,000×, respectively). **(A,D,E)** control (35% H_3_PO_4_); **(B,E,H)**, CPICs+H_3_PO_4_-II; **(C,F,I)**, CPICs+H_3_PO_4_-I. Scale bars represent 10, 1, and 1 µm, respectively. The same results are shown at different magnifications (see each column)..

SEM images were acquired to visually confirm whether CPICs-incorporated H_3_PO_4_ was remineralized. Two mixed solutions of CPICs and H_3_PO_4_ at volume ratios of 1:1 (CPICs + H_3_PO_4_-I) and 1:2 (CPICs + H_3_PO_4_-II) were applied, respectively, to the enamel specimens. The SEM images of the acid-etched enamel revealed a rough enamel rod surface (Figure 3). After treatment with the CPICs-incorporated H_3_PO_4_ solutions, remineralized enamel was confirmed by the presence of numerous Ca–P minerals on the etched enamel surface when compared to the control, and remineralization was found not only between the rods but also in the prism cores (Figures 3B,C). Notably, with a higher concentration of CPICs, the degree of enamel remineralization increased significantly. High-resolution SEM imaging revealed that enamel remineralization occurred with CPICs in a concentration-dependent manner (Figures 3A–C).

**FIGURE 4:**
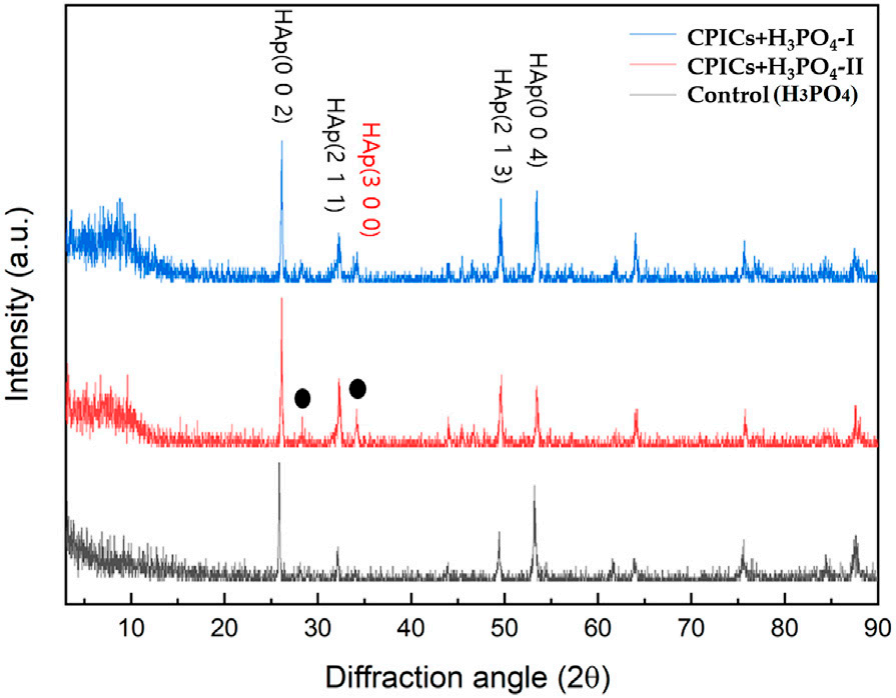
X-ray diffraction (XRD) patterns from the acid-etched enamel specimens (control, CPICs + H_3_PO_4_-II, and CPICs + H_3_PO_4_-I). The black dots and red letter are showing a new HAp peak (ICDD 98–000–1706) that revealed the degree of enamel remineralization increased.

The XRD results identified the HAp peak (ICDD 98–000–1706) for all specimens. However, a new HAp peak was also formed in the CPICs + H_3_PO_4_-I and CPICs + H_3_PO_4_-II specimens. These results revealed that the degree of enamel remineralization increased (Figure 4).

**FIGURE 5:**
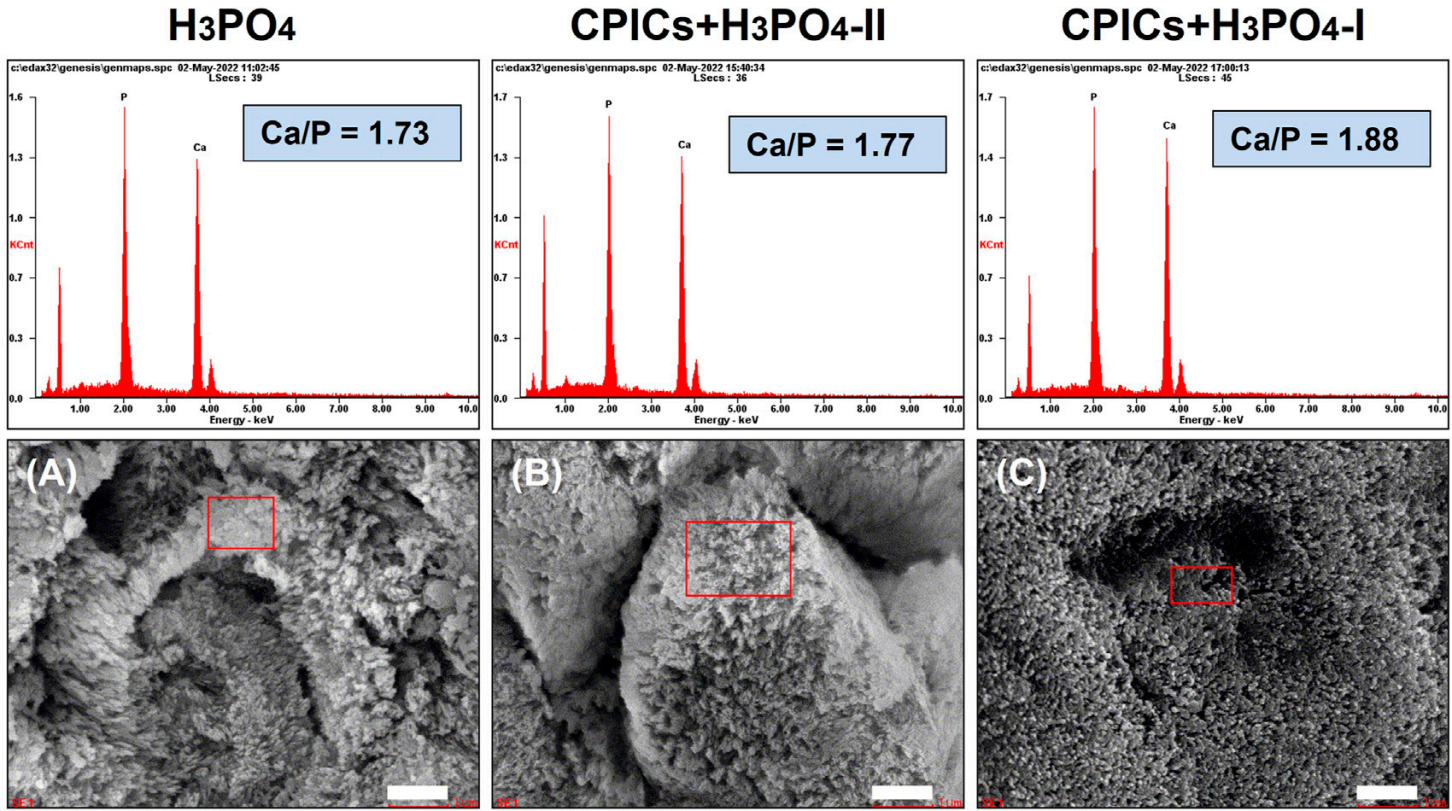
Scanning electron microscope (SEM) images and energy dispersive X-ray spectrometer (EDX) spectra for the acid-etched enamel specimens (A, control; B, CPICs + H_3_PO_4_-II; C, CPICs + H_3_PO_4_-I). Each scale bar represents 1 μm and each image was magnified ×15,000.

The EDX spectra revealed that the main elements in the remineralized layer were calcium, phosphate, and oxygen (Figure 5). The average Ca/P molar ratio increased in a concentration-dependent manner from 1.73 H_3_PO_4_ to 1.88 CPICs-H_3_PO_4_-I.

**FIGURE 6:**
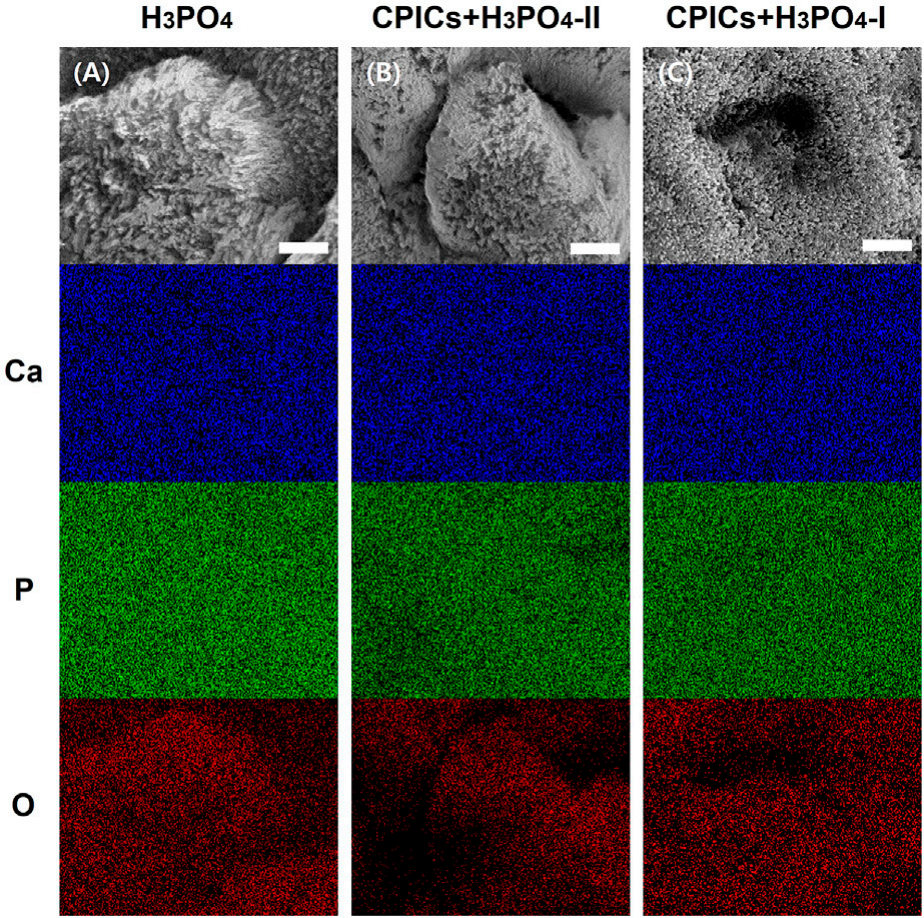
Scanning electron microscope-energy dispersive X-ray spectrometer (SEM-EDX) element mapping for the Ca, P, and O elements on the acid etched enamel specimens (A, control; B, CPICs + H_3_PO_4_-II; C, CPICs + H_3_PO_4_-I). Each scale bar represents 1 μm and each image was magnified ×15,000.

SEM-EDX mapping for all the chemical elements on the surface of the H_3_PO_4_-etched enamel and the CPICs-H_3_PO_4_-II and CPICs-H_3_PO_4_-I-etched enamel samples (Figure 6) confirmed the relatively uniform distribution of O. Figure 6 represents an increased concentration of Ca and a decreased elemental concentration of P in a CPICs concentration-dependent manner.

### 3.2 Phase II: comparison of shear bond strength values and adhesive remnant index scores both before and after thermocycling

**FIGURE 7:**
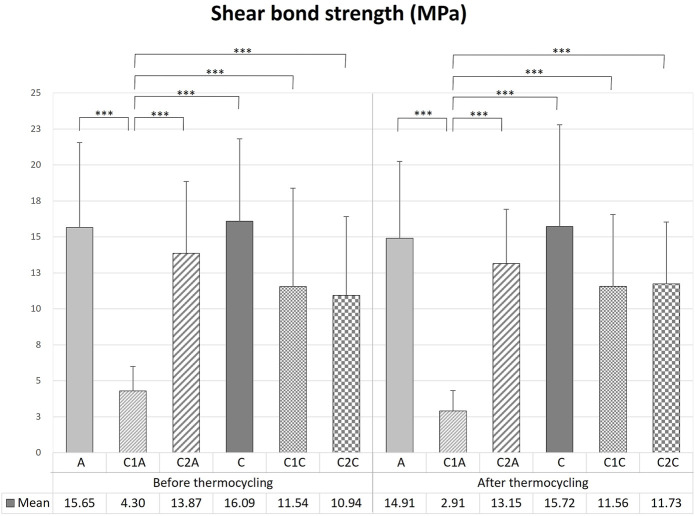
Comparison of the shear bond strength of bracket debonding before and after 10,000 thermocycles. This figure presents the data in Table 1. Error bars represent the standard deviation of the mean. *** indicates significance at *p* < 0.001.

**FIGURE 8:**
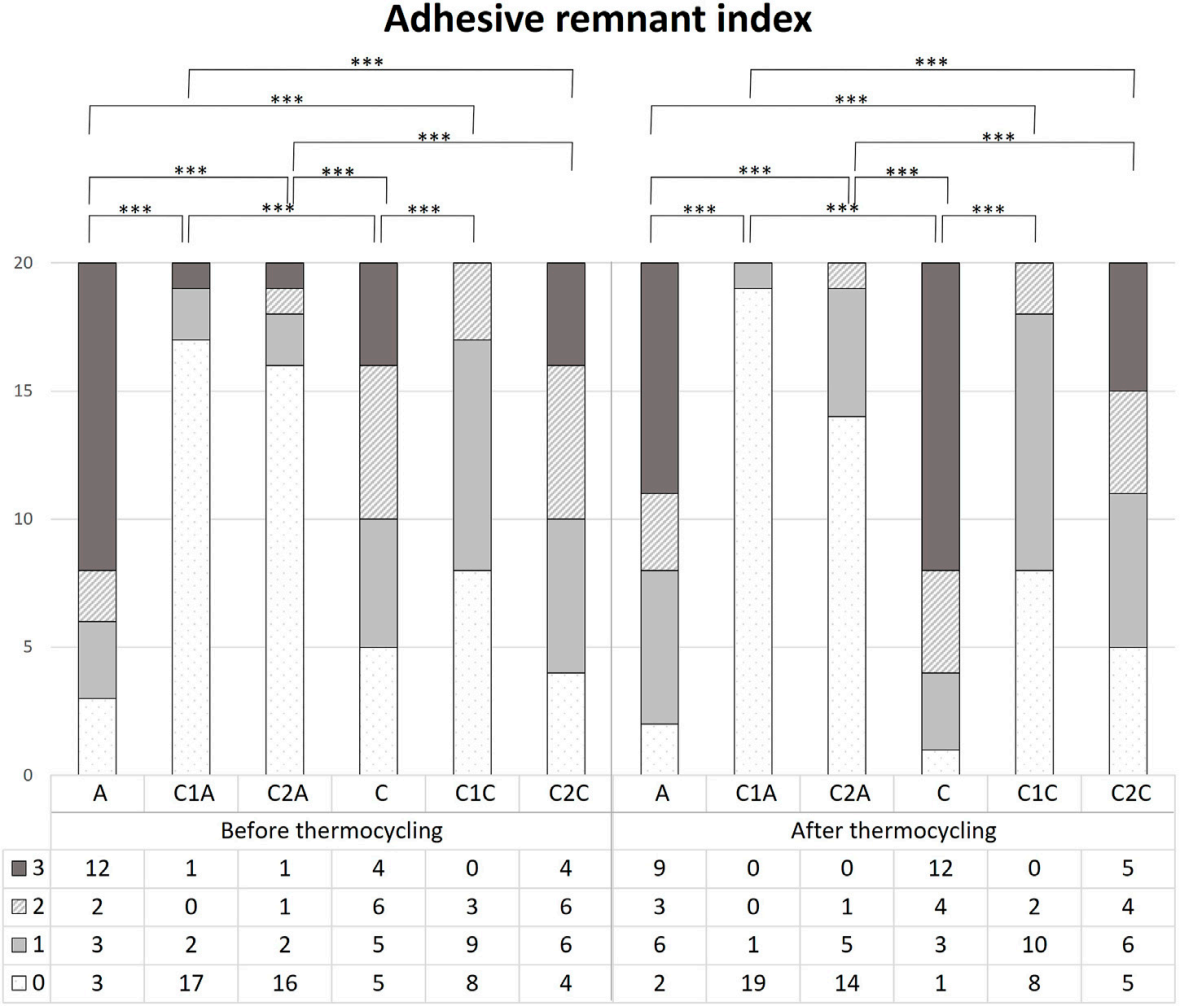
Comparison of the adhesive remnant index scores before and after 10,000 thermocycles. This figure presents the data in Table 3. *** indicates significance at *p* < 0.001.

There was no significant difference in the mean SBS values among the orthodontic resin groups before TC (Tables 1, 2; Figure 7), except for the C1A group (*p* < 0.001). The C1A group had the lowest mean SBS and ARI scores (Tables 3, 4; Figure 8), indicating minimal adhesion.

**TABLE 1:** Comparison of the shear bond strength values for the bracket debonding before and after 10,000 thermocycles.

Group	SBS (MPa)	Range	Statistics	Post-hoc test with Bonferroni correction
Before thermocycling^b^				
A, 35% H_3_PO_4_ + conventional orthodontic resin^a^	20 15.65 ± 5.91	6.91–29.63	Kruskal–Wallis	acdef^b^
			H = 48.734^b^	
			df = 5	
			*p* < 0.001^c^	

C1A, CPICs + H_3_PO_4_-I + conventional orthodontic resin^b^	20	2.50–10.01		b^b^
	4.30 (1.70, 3.68, 5.38)			
C2A, CPICs + H_3_PO_4_-Ⅱ + conventional orthodontic resin^c^	20	4.99–22.05		acdef^b^
	13.87 ± 4.98			
C, 35% H_3_PO_4_ + SAR^d^	20	8.54–27.09		acdef^b^
	16.09 ± 5.72			
C1C, CPICs + H_3_PO_4_-I + SAR^e^	20	7.28–22.30		acdef^b^
	11.54 (6.85, 8.39, 15.23)			
C2C, CPICs + H_3_PO_4_-Ⅱ + SAR^f^	20	6.32–25.20		acdef^b^
	10.94 (5.47, 9.10, 14.57)			
After thermocycling^a^				
A, 35% H_3_PO_4_ + conventional orthodontic resin^a^	20	7.07–25.61	ANOVA	acdef^a^
			F = 18.372^a^	
	14.91 ± 5.34		
			df = 5
C1A, CPICs + H_3_PO_4_-I + conventional orthodontic resin^b^	20	0.93–4.81		b^a^
			*p* < 0.001^c^	
	2.91 ± 1.41			


C2A, CPICs + H_3_PO_4_-Ⅱ + conventional orthodontic resin^c^	20	5.90–21.48		acdef^a^
	13.15 ± 3.77			
C, 35% H_3_PO_4_ + SAR^d^	20	4.10–35.65		acdef^a^
	15.72 ± 7.07			
C1C, CPICs + H_3_PO_4_-I + SAR^e^	20	1.34–22.77		acdef^a^
	11.56 ± 4.99			
C2C, CPICs + H_3_PO_4_-Ⅱ + SAR^f^	20	3.14–19.56		acdef^a^
	11.73 ± 4.31			

Data are presented as n, median (IQR, 1^st^ quartile, 3^rd^ quartile), or mean ± standard deviation.

a The Kruskal–Wallis and Mann–Whitney tests were performed with Bonferroni corrections.

b Statistically significant difference with adjusted *p*-values < 0.05, according to the *post hoc* comparisons with Bonferroni correction.

c One-way analysis of variance (ANOVA) was performed with Tukey’s honestly significant difference multiple comparison tests.

Different letters among the groups both before and after thermocycling indicate a statistically significant difference at adjusted *p*-values < 0.05, according to *post hoc* comparisons with Bonferroni correction.

**TABLE 2:** *Post hoc* comparisons of the shear bond strength values for the bracket debonding before and after 10,000 thermocycles.

Group	A	C1A	C2A	C	C1C	C2C
Before thermocycling^b^						
A	^-^	<0.001^c^	1.000	1.000	1.000	1.000
C1A	<0.001^b^	-	<0.001^c^	<0.001^c^	0.001^c^	0.001^c^
C2A	1.000	<0.001^c^	-	1.000	1.000	1.000
C	1.000	<0.001^c^	<0.001^c^	-	0.728	0.853
C1C	1.000	0.001^c^	1.000	0.728	-	1.000
C2C	1.000	0.001^c^	1.000	0.853	1.000	-
After thermocycling^a^						
A	-	<0.001^c^	0.854	0.995	0.243	0.296
C1A	<0.001^c^	-	<0.001^c^	<0.001^c^	<0.001^c^	<0.001^c^
C2A	0.854	<0.001^c^	-	0.538	0.901	0.936
C	0.995	<0.001^c^	0.538	-	0.075	0.098
C1C	0.243	<0.001^c^	0.901	0.075	-	1.000
C2C	0.296	<0.001^c^	0.936	0.098	1.000	-

Data are adjusted *p*-value.

a The Kruskal–Wallis and Mann-Whitney tests were performed with Bonferroni corrections.

b Statistically significant difference with adjusted *p*-values < 0.05, according to the *post hoc* comparisons with Bonferroni correction.

c One-way analysis of variance (ANOVA) were performed with Tukey’s honestly significant difference multiple comparison tests.

A, 35% H_3_PO_4_ + conventional orthodontic resin; C, 35% H_3_PO_4_ + SAR; C1A, CPICs + H_3_PO_4_-I + conventional orthodontic resin; C2A, CPICs + H_3_PO_4_-II + conventional orthodontic resin; C1C, CPICs + H_3_PO_4_-I + SAR; and C2C, and CPICs + H_3_PO_4_-II + SAR (see Figure 2).

**TABLE 3:** Comparison of the adhesive remnant index scores before and after 10,000 thermocycles.

Group	ARI score				Post-hoc test with Bonferroni correction	Statistics
	0	1	2	3		
Before thermocycling						
A^a^	3	3	2	12	adf	Kruskal–Wallis chi-squared = 42.603, df = 5, *p* < 0.001*
C1A^b^	17	2	0	1	bce	
C2A^c^	16	2	1	1	bce	
C^d^	5	5	6	4	adf	
C1C^e^	8	9	3	0	bcef	
C2C^f^	4	6	6	4	adef	
After thermocycling						
A^a^	2	6	3	9	adf	Kruskal–Wallis chi-squared = 61.987, df = 5, *p* < 0.001^*^
C1A^b^	19	1	0	0	bce	
C2A^c^	14	5	1	0	bce	
C^d^	1	3	4	12	adf	
C1C^e^	8	10	2	0	bcef	
C2C^f^	5	6	4	5	adef	

Different letters among the groups, both before and after thermocycling, indicate a statistically significant difference with an adjusted *p*-value < 0.05, according to *post hoc* comparisons with Bonferroni correction.

A, 35% H_3_PO_4_ + conventional orthodontic resin; C, 35% H_3_PO_4_ + SAR; C1A, CPICs + H_3_PO_4_-I + conventional orthodontic resin; C2A, CPICs + H_3_PO_4_-II + conventional orthodontic resin; C1C, CPICs + H_3_PO_4_-I + SAR; and C2C, and CPICs + H_3_PO_4_-II + SAR (see Figure 2). There was a statistically significant difference in the ARI scores before and after TC. Adhesive remnant index (ARI) scores were as follows: 0, no adhesive left on the tooth; 1, less than half of the adhesive left on the tooth; 2, more than half of the adhesive left on the tooth; and 3, all adhesive left on the tooth with a distinct impression of the bracket mesh.

**TABLE 4:** *Post hoc* comparisons of the adhesive remnant index scores before and after 10,000 thermocycles.

Group	A	C1A	C2A	C	C1C	C2C	
Before thermocycling							
A	^-^	<0.001^a^	<0.001^a^	1.000	0.014^a^	1.000	
C1A	<0.001^a^	-	1.000	0.005^a^	0.961	0.003^a^	
C2A	<0.001^a^	1.000	-	0.016^a^	1.000	0.008^a^	
C	1.000	0.005^a^	0.016^a^	-	1.000	1.000	
C1C	0.014^a^	0.961	1.000	1.000	-	0.838	
C2C	1.000	0.003^a^	0.008^a^	1.000	0.838	-	
After thermocycling							
A	-	<0.001^a^	<0.001^a^	1.000	0.031^a^	1.000	
C1A	<0.001^a^	-	1.000	<0.001^a^	0.305	0.001^a^	
C2A	<0.001^a^	1.000	-	<0.001^a^	1.000	0.033^a^	
C	1.000	<0.001^a^	<0.001^a^	-	0.001^a^	0.430	
C1C	0.031^a^	0.305	1.000	0.001^a^	-	1.000	
C2C	1.000	0.001^a^	0.033^a^	0.430	1.000	-	

Data are adjusted *p*-value.

a Statistically significant difference with an adjusted *p*-value < 0.05, according to *post hoc* comparisons with Bonferroni correction.

Bracket debonding was also performed after all groups were subjected to 10,000 thermocycles. Like the results prior to TC, there were no significant differences in the mean SBS values among the orthodontic resin groups (Tables 1, 2; Figure 7) after TC, except for the C1A group (*p* < 0.001). The mean SBS values recorded before TC tended to decrease after TC in all groups, but the difference was not significant (*p* > 0.05).

Before TC, the most notable difference in the groups using conventional orthodontic binding resin was that the ARI score tended to increase in control group A but decreased in groups treated with CPICs-incorporated H_3_PO_4_ (C1A and C2A) (Tables 3, 4; Figure 8) (*p* < 0.001). Notably, the C1A group presented very little adhesion and a low ARI score, whereas the C2A group had a low ARI score while maintaining a similar level of SBS values to the control group (*p* < 0.001). There were no significant differences in SBS values ​​and ARI scores among the three groups using SAR, namely, the C, C1C, and C2C groups.

After TC, significant differences were noted in ARI scores between the control and CPICs-incorporated H_3_PO_4_ groups, regardless of the use of conventional orthodontic resin or SAR. The control group had a high ARI score, and in comparison, the CPICs-incorporated H_3_PO_4_ groups had relatively lower ARI scores (Tables 3, 4; Figure 8). The C1A group had very low adhesion and low ARI scores, showing the same tendency as before TC. The C2A and C1C groups had lower ARI scores while maintaining a similar level of SBS values ​​to the control group (*p* < 0.001).

The tendency in the groups before and after TC was almost the same, except in group C. This group had significantly higher ARI scores after TC than those before TC (*p* < 0.001).

**FIGURE 9:**
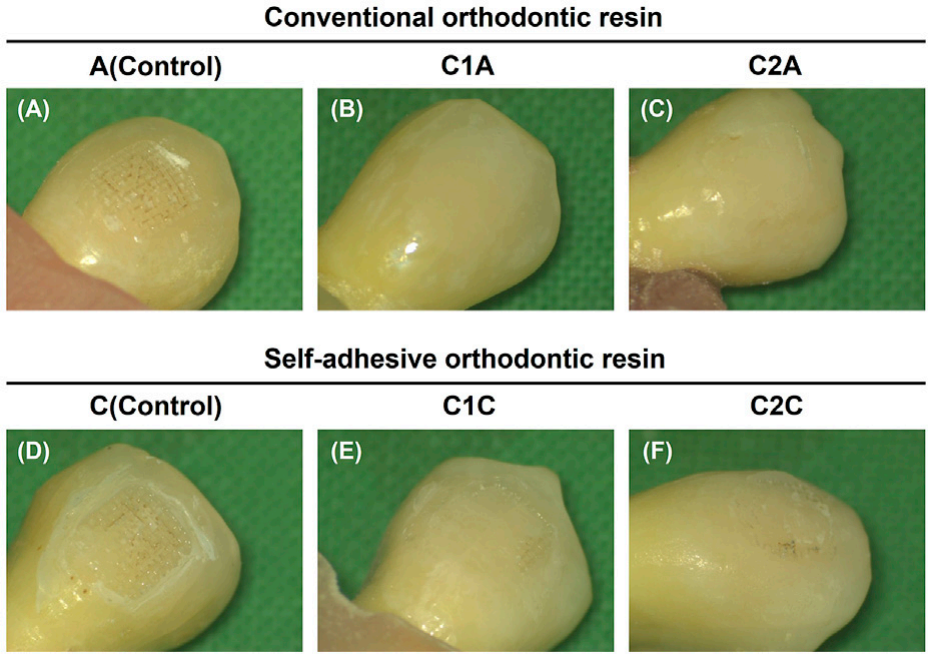
Images showing adhesive remaining on the enamel surface. The adhesive remained after the measurement of the shear bond strength but before thermocycling. **(A)**, 35% H_3_PO_4_ + conventional orthodontic resin; **(B)**, CPICs-incorporated H_3_PO_4_ solution-I + conventional orthodontic resin; **(C)**, CPICs-incorporated H_3_PO_4_ solution-II + conventional orthodontic resin; **(D)**, 35% H_3_PO_4_ + self-adhesive orthodontic resin; **(E)**, CPICs-incorporated H_3_PO_4_ solution-I + self-adhesive orthodontic resin; **(F)**, CPICs-incorporated H_3_PO_4_ solution-II + self-adhesive orthodontic resin.

The microscopic observations of the adhesive remaining after the SBS measurement but before TC reflect the contents in Table 3 and Table 4 and Figure 8 (Figure 9). The control groups A (Figure 9A) and C (Figure 9D) had the largest amount of remnant adhesive, whereas the groups treated with CPICs-incorporated H_3_PO_4_ had relatively less remnant adhesive (Tables 3, 4). Moreover, the most striking difference was the gloss of the enamel surface when comparing the groups using conventional orthodontic bonding resin and SAR, except for the C1A group (Figure 9B), in which the bracket fell off because the minimum bonding force was not present. The groups treated with conventional orthodontic bonding resin (Figures 9A–C) showed a smooth and lucent surface, while those treated with SAR (Figures 9D–F) showed a rough and opaque surface.

### 3.3 Phase III: SEM images of the enamel surfaces after bracket debonding

**FIGURE 10:**
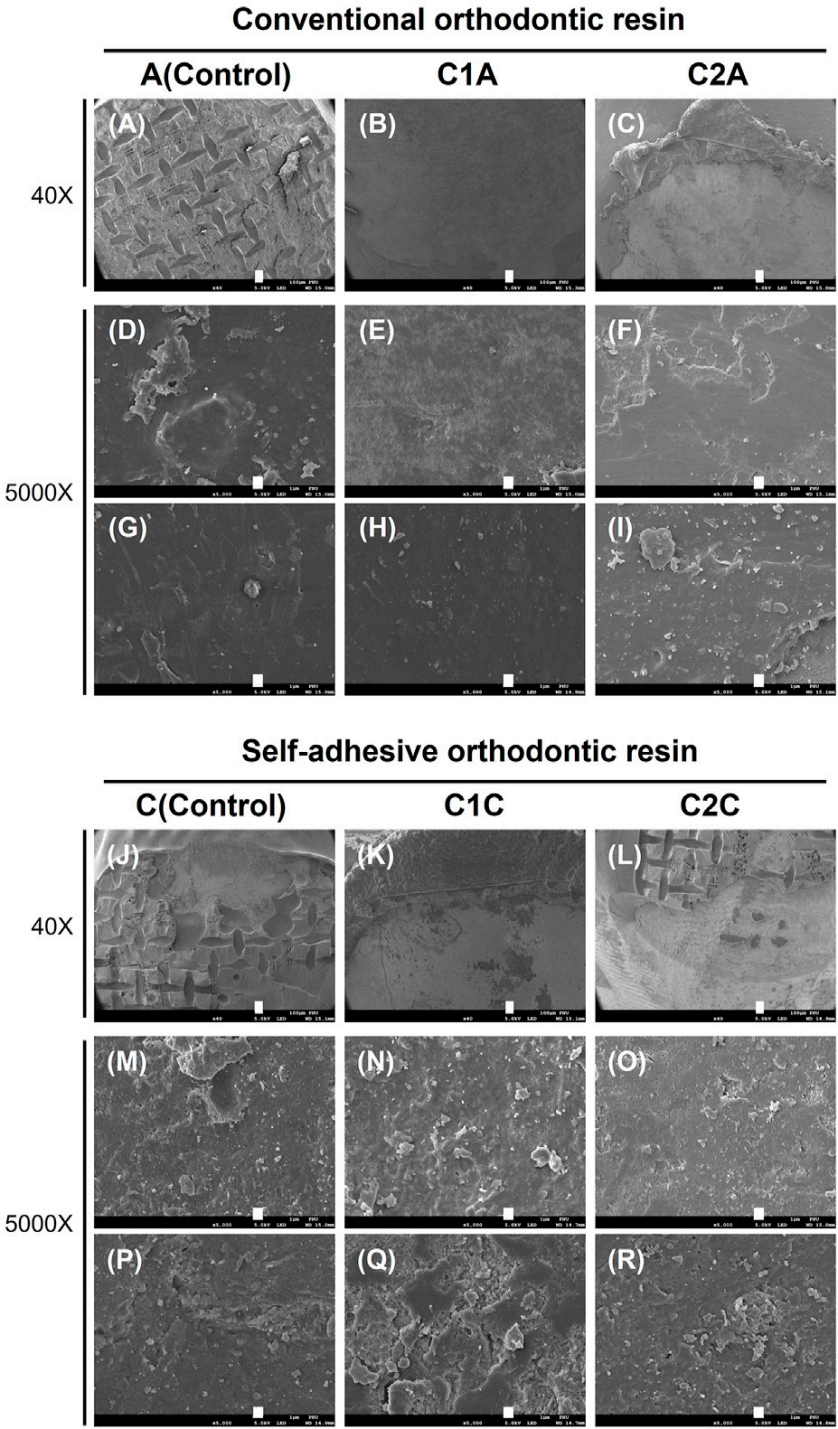
SEM images showing the adhesive remaining on the enamel surface. These images correspond to the SEM images for the tooth specimens shown in Figure 9. Each row represents a different magnification (40× and 5,000×, respectively). Each scale bar represents 100 and 1 µm, in that order. **(A,D,G)**, 35% H_3_PO_4_ + conventional orthodontic resin; **(B,E,H)**, CPICs-incorporated H_3_PO_4_ solution-I + conventional orthodontic resin; **(C,F,I)**, CPICs-incorporated H_3_PO_4_ solution-II + conventional orthodontic resin; **(J,M,P)**, 35% H_3_PO_4_ + self-adhesive orthodontic resin; **(K,N,Q)**, CPICs-incorporated H_3_PO_4_ solution-I + self-adhesive orthodontic resin; **(L,O,R)**, CPICs-incorporated H_3_PO_4_ solution-II + self-adhesive orthodontic resin.

Along with the SBS and ARI values, medical microscopy (Figure 9) and SEM images (Figure 10) were obtained to determine the clinical differences observed on the enamel surface immediately after bracket debonding.

The results in Figure 9 are directly reflected in the SEM images of Figure 10. Group A had the highest ARI scores (Table 3; Figure 8) among the groups using conventional orthodontic resin, and most of the SEM images showed the area where the adhesive remained, and there were hardly any areas with only the enamel surface (Figure 10A). The other observation site on the tooth surface without adhesive (Figures 10D,G) presented a relatively smooth surface when compared to group C (Figures 10M,P).

Furthermore, the surface of the C2A group showed particles and several layers of structures that looked like plates when compared to the surfaces of the A and C1A groups (Figures 10C,F,I). In the C1C and C2C groups (Figures 10N,Q,O,R), a remarkably rough surface was observed when compared to that in group C (Figures 10M,P). In particular, the C1C group showed many more particle-like structures and multiple layer structures, such as contour lines (Figures 10N,Q), when compared to the C2C group (Figures 10O,R).

Differences in the roughness of the enamel surface and number of particles were thus observed between the groups using conventional orthodontic resin (Figures 10A–I) and using SAR (Figures 10J–R). These differences were most pronounced in the C1A, C2A, C1C, and C2C groups treated with CPICs-containing H_3_PO_4_ solutions.

### 3.4 Phase IV: enamel etching and roughness measurements after treatment with H_3_PO_4_, CPICs + H_3_PO_4_-I, and CPICs + H_3_PO_4_-II

**FIGURE 11:**
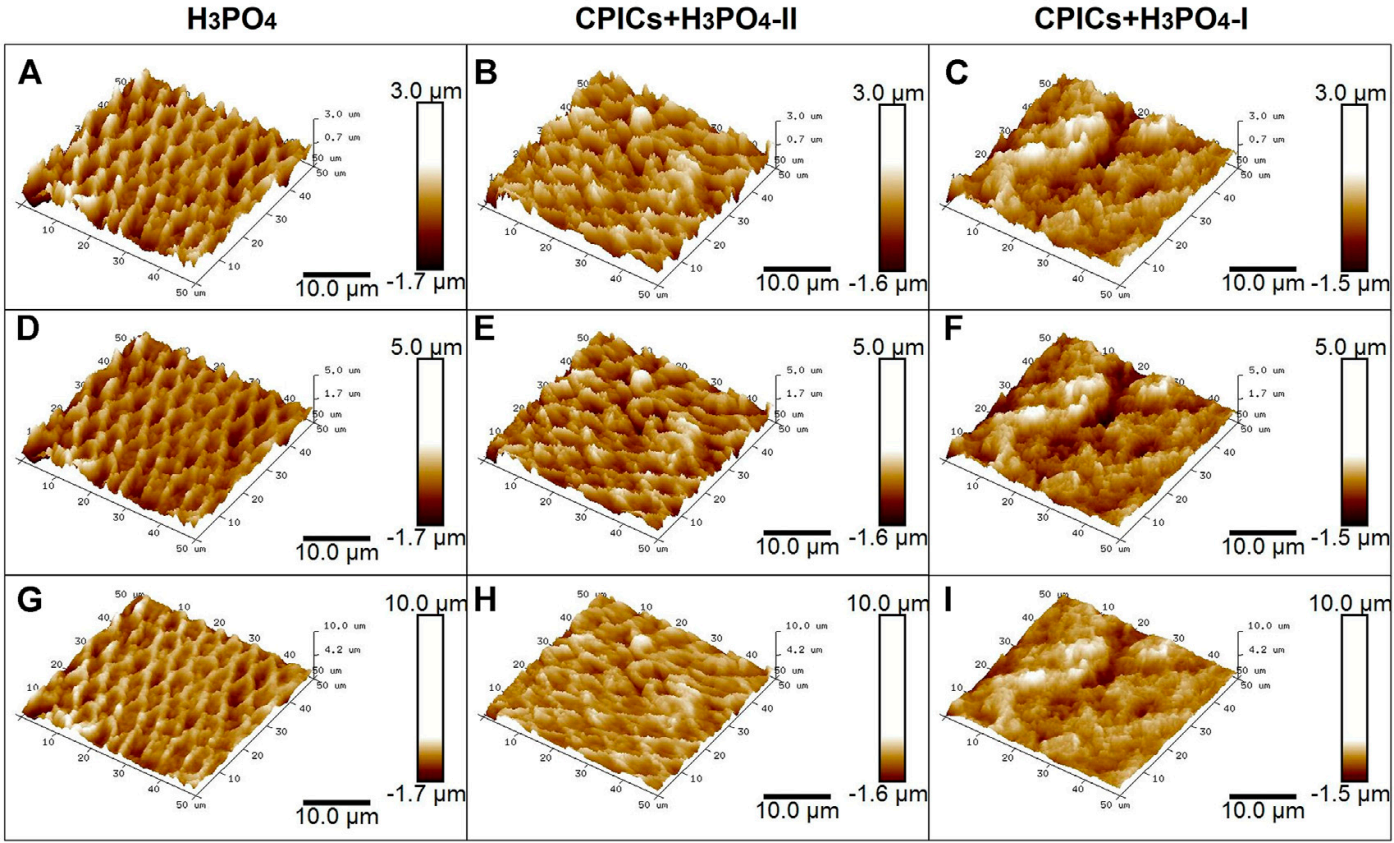
Atomic force microscopy images showing the acid-etched enamel. Each row represents a different range of measurement. The same results were obtained for different measurement ranges (see each column). **(A,D,G)**, control (35% H_3_PO_4_); **(B,E,H)**, CPICs+H_3_PO_4_-II; **(C,F,I)**, CPICs+H_3_PO_4_-I.

AFM images were used to determine the roughness of CPICs-incorporated H_3_PO_4_-treated enamel surfaces (Figure 11). AFM images of enamel surfaces treated with 35% H_3_PO_4_ (control group) revealed a regular and rough surface for enamel rods (Figures 11A,D,G). After treatment with the CPICs-incorporated H_3_PO_4_ solutions, the levels of Ca–P increased on the etched enamel surface when compared to the control group (see Figure 6). Notably, with increasing concentrations of CPICs, the degree of enamel roughness changed (Figures 11A,–C).

**FIGURE 12:**
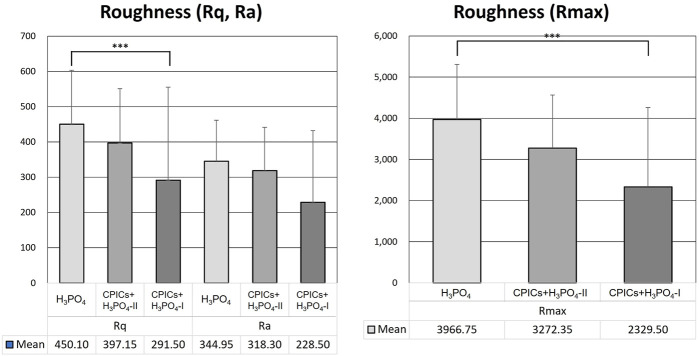
Roughness values of acid-etched enamel. This figure presents the data in Table 5. Error bars represent the standard deviation of the mean. *** indicates significance at *p* < 0.017.

Table 5 and Figure 12 show the roughness values in Figure 11 as measured values. There were no differences between the three groups in terms of their Ra values. However, significant differences were observed in the Rq and Rmax values. The H_3_PO_4_ group had the highest Rq and Rmax values, followed by the CPICs + H_3_PO_4_-II and CPICs + H_3_PO_4_-I groups. However, there was a statistically significant difference between the H_3_PO_4_ and CPICs + H_3_PO_4_-I groups, but the CPICs + H_3_PO_4_-II group did not differ significantly from the other two groups.

**TABLE 5:** Roughness values for the acid-etched enamel.

Group	Rq (nm)	Statistics	Ra (nm)	Statistics	Rmax (nm)	Statistics
H_3_PO_4_	20	Kruskal-Wallis chi-squared = 6.966, df = 2, *p* = 0.031∗	20	Kruskal–Wallis chi-squared = 5.878, df = 2, *p* = 0.053	20	Kruskal–Wallis chi-squared = 9.179, df = 2, *p* = 0.010^b^
	450.1 ± 153.37^a^		344.95 ± 116.89		3,966.75 ± 1,339.03^a^	
CPICs + H_3_PO_4_-II	20		20		20	
	397.15 ± 153.86	df = 2,	318.30 ± 123.32		3,272.35 ± 1,290.20	
CPICs + H_3_PO_4_-I	20	*p* = 0.031^b^				
	291.50 (263.50, 190.75, 454.25)^a^		20		20	
			228.50 (203.25, 147.25, 350.50)		2,329.50 (1933.50, 1795.25, 3,728.75)^a^	

Data are presented as n, median (IQR, 1^st^ quartile, 3^rd^ quartile), or mean ± standard deviation.

a A statistically significant difference at *p*-values < 0.017 = 0.05/3, according to *post hoc* comparisons with a Bonferroni correction.

b A statistically significant difference at *p*-values < 0.05. Average roughness (Ra) value: arithmetic mean for the peak height and valley depth from a mean line. Root mean square roughness (Rq): height distribution relative to the mean line. Maximum roughness depth (Rmax): value representing isolated profile features.

### 3.5 Phase V: comparison of the microhardness of acid-etched enamel surfaces

Microhardness was measured after experimental etchant application to determine whether the enamel remineralization observed in Figures 3–6 affected microhardness. H_3_PO_4_ solutions containing CPICs were applied to the enamel specimens and washed thoroughly.

**FIGURE 13:**
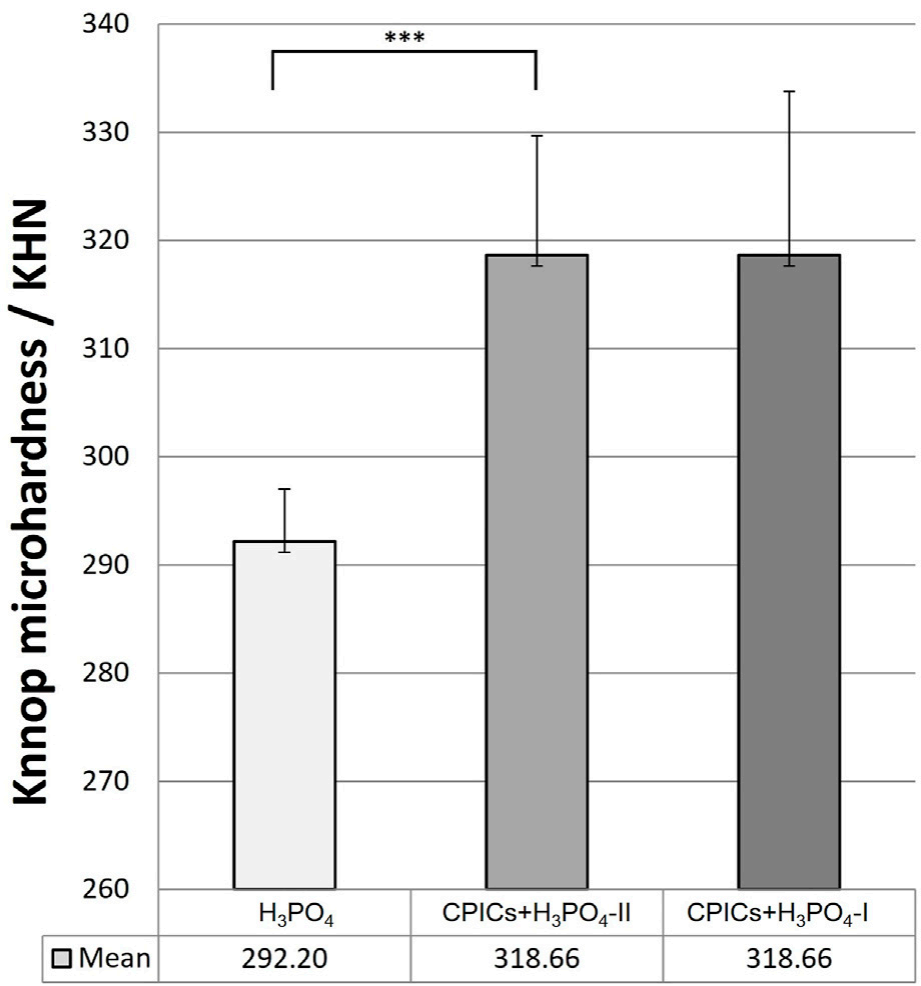
Microhardness scores for the acid-etched enamel. This figure presents the data in Table 6. Error bars represent the standard deviation of the mean. *** indicates significance at *p* < 0.017.

Microhardness measurements are presented in Table 6 and Figure 13. There were significant differences in the mean microhardness values between the two groups (*p* < 0.017). It was observed that the microhardness significantly increased in the group, in which the CPICs-H_3_PO_4_-II solution was applied when compared to the control group where only H_3_PO_4_ was applied. Despite the concentration-dependent remineralization observed in Figure 3, the microhardness recorded was rather high for CPICs + H_3_PO_4_-II, and there was no significant difference between CPICs + H_3_PO_4_-II and CPICs + H_3_PO_4_-I.

**TABLE 6:** Microhardness scores for the acid-etched enamel.

Group	Knoop hardness	Range	Statistics
H_3_PO_4_	18	262.80–297.60	Kruskal–Wallis chi-squared = 9.179, df = 2, *p* = 0.010^b^
	292.20 (4.83, 290.70, 320.20)^a^		
CPICs + H_3_PO_4_-II	18	305.50–336.90	
	318.66 ± 11.00^a^		
CPICs + H_3_PO_4_-I	18	284.00–327.80	
	318.66 ± 15.10		

Data are presented as n, median (IQR, 1^st^ quartile, 3^rd^ quartile), or mean ± standard deviation.

a Statistically significant differences at *p* < 0.017 = 0.05/3, according to *post hoc* comparisons with Bonferroni correction.

b A statistically significant difference at *p*-values < 0.05.

## 4 Discussion

In this study, the effects of etchants with H_3_PO_4_ containing Ca–P ion clusters (CPICs) on enamel were evaluated during bracket debonding. This study aimed to investigate whether (Latta and Radniecki, 2020) CPICs-containing experimental etchants could achieve enamel remineralization and (Cengiz and Ünal, 2019) if there was a difference in the SBS and ARI scores between the CPICs-incorporated H_3_PO_4_ and H_3_PO_4_. First, experimental etchants containing CPICs achieved enamel remineralization. This was verified using SEM/EDX, element mapping, XRD, and AFM. Also, the roughness and microhardness of the enamel surface were better in the group remineralized by the experimental etchant containing CPICs. Second, when the C2A group under the conditions before thermocycling and the C2A and C1C groups under the conditions after thermocycling, that is, the experimental etchant group containing CPICs, compared to the H_3_PO_4_ group used as a control, where the SBS did not decrease and left less adhesive residue.

To investigate the effects of the CPICs-H_3_PO_4_ etchant after bracket debonding, the SBS and ARI scores before and after thermocycling were measured. Only the C1A group showed a significant difference in the results among the 12 groups (Tables 1, 2; Figure 7), for which the SBS was measured. The mean SBS values of the C1A were 4.98 MPa before TC and 3.24 MPa after TC. These were much lower than the minimum SBS of approximately 6–8 MPa, which can maintain coupling with the magnetic force mentioned by Reynolds (1975). Therefore, the combination of conventional orthodontic resin and CPICs-H_3_PO_4_-I had the lowest levels of clinical applicability. The ARI scores show that for most groups, except for the C1A group where SBS did not reach the minimum value, there were no significant differences before and after TC (Tables 3, 4; Figure 8). However, the C2A group had significantly lower ARI scores when compared with the control group before and after TC. C2A was the only group with the same SBS level as the control group, and most of the samples showed an ARI score close to 0 (Tables 1, 2). The groups using SAR, C1C, and C2A had the lowest ARI score at after TC. These results suggest that when CPICs are included in the etchant they can lower the probability of the remaining adhesive. After measuring the SBS and ARI values, images of the enamel surface before TC were acquired using a medical microscope (Figure 9) and SEM (Figure 10) to confirm the clinical differences observed on the enamel surface immediately after bracket debonding. The light and electron microscopy results visually reproduced the SBS and ARI measurements. The roughness of the enamel surface was greater in the group treated with self-adhesive resin (Figures 10J–R) than in the group treated with conventional orthodontic resin (Figure 10A–I). When the C1A group, which had no clinical significance, was excluded, consistent smoothness was observed regardless of the magnification of the surface of the C2A group, which had the best results. Based on these results, it was found that the H_3_PO_4_ group containing CPICs had a much lower ARI than the control group, while maintaining the SBS at a similar level to that of the control.

This study also investigated whether the enamel surface was remineralized when CPICs or H_3_PO_4_ containing CPICs were used. First, an experimental etchant was applied to the enamel surface, and the enamel surface was observed using an SEM. The CPICs + H_3_PO_4_-I and CPICs + H_3_PO_4_-II groups were remineralized with numerous Ca–P mineral particles on the enamel rod and prism core when compared to the control group (Figure 3). For a more objective analysis, each specimen was analyzed by XRD (Figure 4), EDX spectroscopy (Figure 5), and SEM-EDX elemental mapping (Figure 6). These results also show that remineralization occurs on the surface etched with CPICs-H_3_PO_4_ when compared to the H_3_PO_4_ control that does not occur remineralization at all. The AFM images (Figure 11) show the same pattern as the SEM images (Figure 3). Among the roughness measurements obtained from the AFM image, a decrease in the concentration-dependent manner of the CPICs at Rq and Rmax was observed (Table 5; Figure 12). Statistical analysis revealed that the measured values in the CPICs + H_3_PO_4_-I group were significantly different from those in the H_3_PO_4_ group (*p* < 0.017). It was questioned whether the observed remineralization on the enamel surface would affect the microhardness. Surprisingly, the CPICs + H_3_PO_4_-I and CPICs-H_3_PO_4_-II groups showed significantly higher microhardness values than the control group, and the value was significantly higher in the CPICs + H3PO4-II group (*p* < 0.017). Combining these results with those of the previous C2A group, it was concluded that the adhesive strength of the orthodontic bracket increased, and the residual adhesive decreased when H_3_PO_4_ containing an appropriate amount of CPICs was used. Furthermore, it also helped to prevent enamel damage through remineralization. Thus far, the results have suggested that Ca–P-based etchants could have clinical applicability for surface remineralization while minimizing enamel damage during bracket debonding.

Previously, Kim et al. studied the mechanism of dentin remineralization using a novel type of CPICs published by Shao et al. (Shao et al., 2019; Kim et al., 2020). Enamel remineralization through CPICs-H_3_PO_4_ application (Figure 3) was found to have the same pattern as dentin remineralization when the CPICs were treated separately after etching. Enamel remineralization using the application of CPICs-H_3_PO_4_ was also demonstrated in a recently published study by Lee et al. Considering that the pH of H_3_PO_4_ is quite low (approximately 0.8), it is very interesting that CPICs can be remineralized even in H_3_PO_4_ solutions. Hints regarding the behavior of Ca–P-based materials with respect to pH can be obtained from the work of Ibrahim et al. (2019). Etching the enamel with H_3_PO_4_ creates a slight buffering action on the surface while simultaneously dissolving calcium in the enamel. Ibrahim et al. explained that when Ca–P-based materials are included in the etching solution, phosphate is buffered, causing the re-precipitation of Ca–P and simultaneously limiting the demineralization of the enamel by acidic solutions. Another study by Ibrahim et al. using a Ca–P-based etchant also found that enamel conditioned with an acidic Ca–P paste (β-tricalcium phosphate and monocalcium phosphate monohydrate powders mixed with 37% H_3_PO_4_ solution) achieved adequate bond strength with minimal or no adhesive residue or enamel damage (Ibrahim et al., 2020). In a study by Ibrahim et al., the pH was increased from 0.8 to 1.4 after mixing Ca–P powder in 35% H_3_PO_4_, and this resulted in a less aggressive etchant that prevented enamel demineralization through a buffering effect and improved adhesion (Ibrahim et al., 2019). Surprisingly, the pH values of CPICs-H_3_PO_4_-I and CPICs-H_3_PO_4_-II used in this study were 0.7 and 1.7, respectively, suggesting a possible buffering effect of the CPICs-H3PO4 etchant.

**FIGURE 14:**
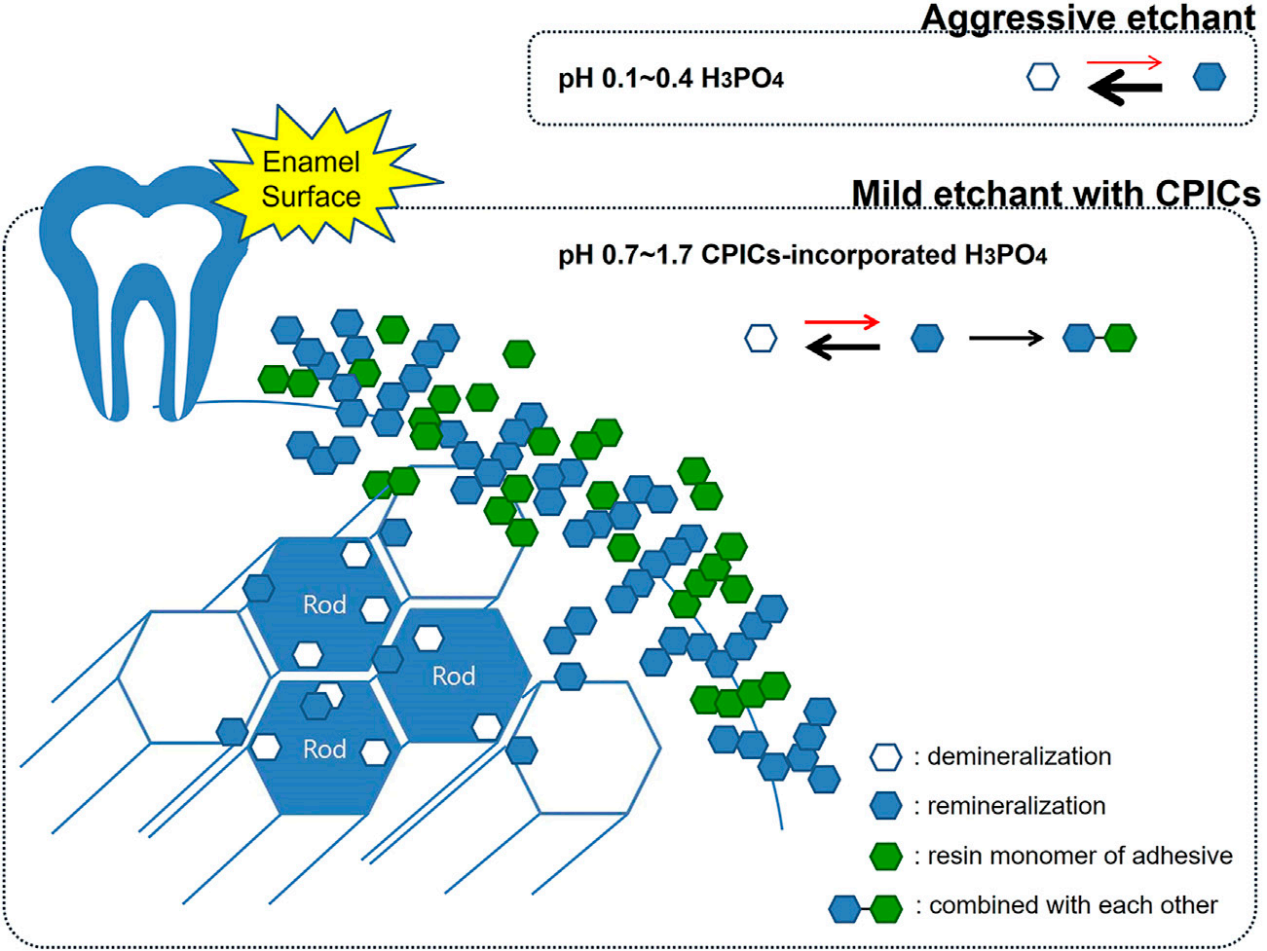
Schematic representation showing enamel remineralization induced by the calcium phosphate ion clusters-incorporated phosphoric acid solution. Using a Ca–P buffering to create a less aggressive etchant not only protects the enamel but also lowers enamel demineralization. The crystals generated by the remineralization of Ca–P were found to combine with the adhesive to improve the bonding strength and protect the enamel when the bracket is removed.

Furthermore, the enamel remineralization effect of a Ca–P-based etchant can be considered in relation to the conventional “crystal bonding” concept. This concept was developed by Smith (1968); Maijer and Smith (1986), wherein crystal bonding has been proposed as an alternative technique to etch the enamel for retention of an adhesive by growing crystals on the enamel surface. As this method does not reach the bonding strength of conventional acid etching using PA and is technique sensitive, it is not currently used clinically (Read et al., 1986). However, the principle of growing sulfate dihydrate crystals using a crystal binding solution based on a mixture of polyacrylic acid and residual sulfate ions and the advantages of this method are consistent with the findings of this study. In the study by Maijer and Smith (1986), the advantages of crystal bonding reduced enamel damage, easy debonding, and reduced amount of adhesive left on the teeth, which are the same as the advantages of the CPICs-incorporated H_3_PO_4_ etching solution. Using a Ca–P buffering to create a less aggressive etchant not only protects the enamel but also lowers enamel demineralization. From the concept of crystal bonding, the idea that newly formed inorganic crystals contribute to the bonding strength was obtained, the crystals generated by the remineralization of Ca–P were found to combine with the adhesive to improve the bonding strength and protect the enamel when the bracket is removed. This was the core mechanism of the Ca–P-integrated etching solution, and it is shown schematically in Figure 14.

Because the results using CPICs + H_3_PO_4_-I were incomplete and that CPICs + H_3_PO_4_-II with a relatively high pH generally outperformed CPICs + H_3_PO_4_-I, it is clear that it will be necessary for identifying appropriate proportion of CPICs and H_3_PO_4_ to work efficiently. This suggests areas for future research regarding CPICs-incorporated in H_3_PO_4_. A limitation of this study is that 20 samples were allocated to each group without prior testing regarding the sample size. Although statistical analysis results are provided, the positive results of this study will be more reliably supported when a larger number of samples are allocated to each group. Despite the limitations of this study, the potential for remineralization with CPICs-H_3_PO_4_ is clearly worthy of further investigation, and this body of research will serve as the basis for the development of clinically safer and more feasible etchants.

## Conclusion

Twelve experimental groups were created using three experimental etchants, two resins, and the before and after thermocycling criteria. Due to measuring the shear bond strength (SBS) and adhesive remnant index (ARI) values for each experimental group, the C2A group under the condition before TC, and the C2A and C1C groups under the condition after TC were shown to decrease the ARI scores without reducing SBS during debonding. Furthermore, after applying the three etching solutions to the enamel surface, the enamel surface was observed using an SEM and AFM, the X-ray diffraction patterns and EDX spectrum measurements were assessed, and elemental mapping was performed. The results showed that the etchant containing CPICs remineralized the enamel according to the concentration gradient, and that the Knoop microhardness increased in CPICs-H_3_PO_4_-II when compared to the control.

## Data Availability

The raw data supporting the conclusion of this article will be made available by the authors, without undue reservation.

## Nomenclature

TC: thermocycling
SBS: shear bond strength
CPICs: calcium phosphate ion clusters
CPICs + H_3_PO_4_-I: CPICs and H_3_PO_4_ in volume ratio of 1:1
CPICs + H_3_PO_4_-II: CPICs and H_3_PO_4_ in volume ratio of 1:2
A: 35% H_3_PO_4_ + conventional orthodontic resin
C: 35% H_3_PO_4_ + self-adhesive orthodontic resin
C1A: CPICs-incorporated H_3_PO_4_ solution-I + conventional orthodontic resin
C2A: CPICs-incorporated H_3_PO_4_ solution-II + conventional orthodontic resin
C1C: CPICs-incorporated H_3_PO_4_ solution-I + self-adhesive orthodontic resin
C2C: CPICs-incorporated H_3_PO_4_ solution-II + self-adhesive orthodontic resin
MMPs: matrix metalloproteinases
